# *Streptomyces lydicus* M01 Regulates Soil Microbial Community and Alleviates Foliar Disease Caused by *Alternaria alternata* on Cucumbers

**DOI:** 10.3389/fmicb.2020.00942

**Published:** 2020-05-15

**Authors:** Mingxuan Wang, Jian Xue, Junjie Ma, Xiaohai Feng, Hanjie Ying, Hong Xu

**Affiliations:** ^1^State Key Laboratory of Materials-Oriented Chemical Engineering, Nanjing Tech University, Nanjing, China; ^2^College of Food Science and Light Industry, Nanjing Tech University, Nanjing, China; ^3^College of Biotechnology and Pharmaceutical Engineering, Nanjing Tech University, Nanjing, China

**Keywords:** plant growth promotion, *Streptomyces lydicus* M01, rhizospheric microbial community, *Alternaria alternata*, reactive oxygen species, disease resistance

## Abstract

Due to the adverse effect on the environment caused by excessive use of chemical fertilizers, the development of sustainable agriculture attracts a growing demand of biological based fertilizers composed of living microorganisms. In this study, an Actinobacteria *Streptomyces lydicus* M01 was isolated from the rhizosphere soil of *Pyrus calleryana*. This strain effectively promoted the plant growth and suppressed a foliar disease caused by *Alternaria alternata* on cucumbers. *S. lydicus* M01 exhibited growth promoting characteristics such as phosphate solubilization, IAA secretion, siderophore and ACC deaminase production. Through Illumina sequencing of the 16S rRNA gene and ITS gene of the soil microbes, we found that the application of *S. lydicus* M01 altered the composition of the microbial community by promoting beneficial groups, including bacteria genera *Pseudarthrobacter*, *Sphingomonas*, *Rhodanobacter*, and *Pseudomonas*, fungi genera *Fusicolla*, *Humicola*, *Solicoccozyma*, and *Paraphaeosphaeria*. Most of these bacteria and eukaryotes exhibit positive effects on growth promotion, such as nutrient accumulation, auxin secretion, abiotic stress alleviation, biological control, or bioremediation. Furthermore, studies on the reactive oxygen species (ROS) level and antioxidants of cucumber leaves revealed that *S. lydicus* M01 treatment reduced the ROS accumulation and increased the activities of antioxidases related with ROS scavenging, which indicated an enhanced disease resistance of cucumbers under biotic stress. Thus, our results suggest that the application of *S. lydicus* M01 can systemically affect plant microbiome interactions and represent a promising sustainable solution to improve agricultural production instead of chemical fertilizers.

## Introduction

Chemically synthesized fertilizers composed of nitrogen, phosphorus, and potassium are being used in large amounts to increase the output in a variety of crop plants. At the same time, their excess utilizations adversely affect the environment, causing pollution to soil, water and air (Galloway et al., 2008; Youssef and Eissa, 2014). Thus, sustainable agriculture attracts a growing demand of bio-based fertilizers composed of living microorganisms alternative to the agro-chemicals. Plants and rhizosphere harbor a variety of microbial cells, and the plant-associated microbes can be classified into beneficial, deleterious and neutral groups on the basis of their effects on plant growth (Beneduzi et al., 2012). Beneficial bacteria that promote plant growth are usually referred to as plant growth-promoting rhizobacteria (PGPR) (Ahmad et al., 2008; Bhattacharyya and Jha, 2012).

Plant growth-promoting rhizobacteria affect plant growth in direct and indirect ways. The direct promotion of plant growth by PGPR entails either providing the plant with a compound that is synthesized by the bacterium, for example, siderophore and phytohormones such as auxin, cytokinin, and gibberellins, or facilitating the uptake of certain nutrients from the environment such as phosphate solubilization, nitrogen fixation, and iron chelation (Glick, 1995; Olanrewaju et al., 2017). The indirect promotion of plant growth occurs when PGPR decrease the detrimental effects of phytopathogenic organisms or induced systemic resistance (ISR) (Raupach et al., 1996; Pieterse et al., 2014).

*Streptomyces* constitute a major clade of the phylum Actinobacteria, these filamentous prokaryotes are ubiquitous in soils and are commonly found in the rhizosphere or inside plant roots. *Streptomyces* are known to rich in secondary metabolites, they produce over two-thirds of the clinically useful antibiotics of natural origin and are highlighted in medical industry (Watve et al., 2001; Bibb, 2013). The applications of *Streptomyces* in agriculture are mainly as biocontrol agents, several biocontrol products from this genus are already being marketed and the discovery of natural products is still ongoing (Yuan and Crawford, 1995; Law et al., 2017). However, as a PGPR, the plant growth promotion effects of *Streptomyces* have not been adequately studied. Some study reported that *Streptomyces* promote plant growth by the producing indole-3-acetic acid (IAA), siderophores and 1-aminocyclopropane-1-carboxylate deaminase (ACCd) to reduce stress in plants (El-Tarabily, 2008; Rungin et al., 2012; Sadeghi et al., 2012). *Streptomyces* reside in rhizosphere and interact with other microorganisms in the microbial community, but little is known on their effects in soil microbial community (Wu et al., 2019). Cucumber (*Cucumis sativus* L.) is one of the most common vegetables worldwide, many studies reported that strains such as *Bacillus subtilis*, *Bacillus amyloliquefaciens*, and *Pseudomonas* spp. affect soil microbial community in the cucumber rhizosphere(Yamamoto-Tamura et al., 2011; Li et al., 2016; Qin et al., 2017), however, as a promising sources of biocontrol agents, how *Streptomyces* affect soil microbial community in cucumber rhizosphere remains unknown.

Leaf blight caused by the fungus *Alternaria alternata* is a common foliar disease in a variety of fruits and vegetables including pears, cucumber, and watermelon. The pathogen also develops during the cold storage of fruits, becoming visible during the marketing period thereby causing large losses (Timmer et al., 2003; Wang et al., 2008). In this study, an Actinobacteria isolated from the rhizosphere soil of Callery pear (*Pyrus calleryana*) in Nanjing Laoshan Forest exhibited strong inhibition effects against *A. alternata*. Based on 16S rRNA gene and phylogenetic analysis, the strain was identified as *S. lydicus* M01. We found that this strain significantly promote the growth of cucumbers without obvious plant pathogen present. The growth promoting mechanisms were evaluated from the perspective of the phytohormone production of *S. lydicus* M01 and its influence on the microbial community in cucumber rhizosphere. In addition, the alleviation of foliar disease caused by *A. alternata* on cucumbers was also evaluated, the reduction of reactive oxygen species (ROS) accumulation and increased antioxidase activities in cucumber leaves indicated indirect effects of *S. lydicus* M01 on plant growth promotion and disease suppression. To our knowledge, this is the first report of using *Streptomyces* to treat a foliar disease on cucumbers and its effects in cucumber rhizosphere microbial community.

## Materials and Methods

### Isolation and Identification of *Streptomyces* Strain

Soil samples were collected from the rhizosphere of Callery pear (*P. calleryana*) in a hillside located in Laoshan Forest, Nanjing, China (32°11′N 118°62′E). For preparation of the soil suspension sample, 5 g of soil was mixed with 45 ml sterile distilled water in a sterile bottle and shaken for 30 min. Then, 200 μl of the suspension was spread on PDA medium supplemented with 20 mg/l nalidixic acid and 50 mg/l cycloheximide. For preliminary classification of bacterial population, morphological features such as colony pigmentation were used. After incubation at 28°C for 21 days, colonies with suspected actinomycetes morphology were selected and further purified on International Streptomyces Project (ISP) 2 medium and stored as glycerol stock (20%, v/v) at −80°C.

The genomic DNA of M01 was extracted from cells grown in potato dextrose broth (PDB) medium at 28°C for 24 h using a bacterial genomic DNA extraction kit (Takara) according to standard protocol. The universal bacterial primers 27F and 1492R (27F: 5′-AGAGTTTGATCCTGGCTCAG-3′; 1492R: 5′-GGTTACCTTGTTACGACTT-3′) were used to amplify the 16S rRNA gene. The PCR reaction volume was 50 μl, which contains 25 μl Taq DNA Polymerase and reaction buffer mix, 1 μl primer forward (10 μM), 1 μl primer reverse (10 μM), 1 μl genomic DNA template and 22 μl nuclease free water. The reaction conditions were 95°C for 5 min, then 30 cycles of 95°C for 30 s and 55°C for 30 s followed by 72°C for 90 s. A final step was performed at 72°C for 5 min. The PCR products were separated and sequenced by Genscript (Nanjing). The assembled 16S rRNA sequences were subjected to a NCBI Nucleotide BLAST. The neighbor-joining tree of the 16S rRNA was generated using MEGA ver. 7 (Saitou and Nei, 1987). Parameters for developing neighbor-joining tree included the use of the Poisson substitution model and a pairwise deletion method to treat gaps in the program.

### Antagonistic Effects of *Streptomyces lydicus* M01 on Pathogens

The antifungal activity of M01 against Leaf blight fungus *A. alternata* was evaluated using Petri dish assays. Briefly, *S. lydicus* M01 was cultured on ISP 2 plate at 28°C for 14 days. The plant pathogen *A. alternata* was cultured on PDA plates at 28°C for 4 days. A square inoculum of *A. alternata* was using sterile scoop and placed on a new PDA plate. At the same time, *S. lydicus* M01 was restreaked on the four sides around the square inoculum with a distance of 2 cm. Plates inoculated with *A. alternata* in the absence of M01 served as control experiments. All samples were incubated at 28°C for 3 days.

#### Determination of Siderophore Production, Phosphate Solubilization and Indole-3-Acetic Acid

Chrome-Azurol S (CAS) agar was used to determine siderophore production qualitatively (Alexander and Zuberer, 1991). Paper disc soaked with the strain culture was placed on the CAS agar and incubated for 3 days. The change of the mixture color around the colonies after incubation indicated the presence of siderophores.

*Streptomyces lydicus* M01 was inoculated on agars containing precipitated tricalcium phosphate to determine phosphate solubilization according to Rao (1999). After 24 h incubation, presence of clear zones around the M01 colonies was used as evidence for positive phosphate solubilization.

*Streptomyces lydicus* M01 grown on ISP 2 agar was inoculated into PDB and incubated at 28°C for 5 days with shaking at 200 rpm, mycelia were removed by filtration. The IAA content was determined based on the method described previously (Patten and Glick, 2002). One milliliter supernatant of the *S. lydicus* M01 culture was mixed with 4 ml of Salkowski’s reagent vigorously and incubated at room temperature for 25 min absorbance at 535 nm. The concentration of IAA was determined by absorbance at 535 nm and comparison with standard curve.

### Determination of ACC Deaminase Activity

*Streptomyces lydicus* M01 grown on ISP 2 agar was streaked on minimal medium agar free of nitrogen (MM) supplemented with either 3 mM ACC or 2 g/L^–1^ (NH_4_)_2_SO_4_. The agar was incubated at 28°C in the dark. Growth on MM agar supplemented with ACC indicates ACC deaminase activity (Jaemsaeng et al., 2018).

### Pot Experimental Design and Rhizosphere Soil Sampling

The *S. lydicus* M01 culture was grown in PDB on a rotary shaker (200 rpm) for 4 days at 28°C. The cell suspension (2 × 10^8^ CFU/ml) was used as inoculum for the pot experiment.

Cucumber Seed Shaoshi No. 1 was purchased from the Qingxian Aisen Vegetable Seed Development Center. The seeds were sown in plastic pots contained a mixed substrate of vermiculite, peat soil, and perlite at a ratio of 1:1:0.5 (v:v:v). Then 50, 100, and 150 ml of the *S. lydicus* M01 cell suspensions were applied as a root drench. Equivalent volume of sterile PDB was applied as control treatments (CKs) and half-strength Hoagland’s nutrient solution was supplied every week. Three plants were planted per pot. The growth of cucumber seedlings were assessed 21 days after inoculation. The rhizosphere soil samples of cucumber seedlings were collected by vigorously shaking the roots from the 100 ml *S. lydicus* M01 cell suspension treatment and control for high-throughput sequencing. The shoot length, root length, and fresh weights of cucumber seedlings were measured. Each experiment was repeated three times.

For the investigation of *S. lydicus* M01’s effects on foliar disease caused by *A. alternata*, 8-week-old plants were sprayed with a fresh spore suspension of *A. alternata* at 1 × 10^8^ CFU/ml using a hand atomizer. The plants were maintained in a light growth chamber and were supplied with half-strength Hoagland’s nutrient solution every 7 days. Disease incidence was assessed 30 days after inoculation with *A. alternata*. The disease severity index was rated 0–4 (0 = healthy; 1 = less than 25% of leaves wilted; 2 = 25–50% of leaves wilted; 3 = 50–75% of leaves wilted; 4 = 75–100% of leaves wilted). The disease index was calculated using the formula: Disease index = [Σ(rating × number of plants rated)/(total number of plants × highest rating)] × 100 (Shi et al., 2017). Cucumber leaves were frozen using liquid nitrogen and stored at −20°C for later determination of antioxidases activities.

### Soil DNA Extraction, PCR Amplification and Illumina Sequencing

Microbial community genomic DNA was extracted from soil samples using the E.Z.N.A^®^ soil DNA Kit (Omega Bio-tek, Norcross, GA, United States). The hypervariable region V3–V4 of the bacterial 16S rRNA gene were amplified with primer pairs 338F (5′-ACTCCTACGGGAGGCAGCAG-3′) and 806R (5′-GGACTACHVGGGTWTCTAAT-3′) whilst the fungal ITS sequence gene were amplified with primer pairs ITS1F (5′-CTTGGTCATTTAGAGGAAGTAA-3′), and ITS2R (5′-GCTGCGTTCTTCATCGATGC-3′). The PCR amplification was performed as follows: initial denaturation at 95°C for 3 min, followed by 27 cycles at 95°C for 30 s, 55°C for 30 s, 72°C for 45 s, and final extension at 72°C for 10 min, end at 4°C. PCR reactions were performed in triplicate. PCR products was purified and quantified using Quantus^TM^ Fluorometer (Promega, United States).

High-throughput sequencing was carried out on the Illumina MiSeq platform by Majorbio Bio-Pharm Technology Co., Ltd. (Shanghai, China). The raw 16S rRNA gene sequencing reads were demultiplexed, quality-filtered by Trimmomatic and merged by FLASH. The quality trimming process are as follows: (1) The 300 bp reads were truncated at any site receiving an average quality score <20 over a 50 bp sliding window, discarding the truncated reads that were shorter than 50 bp. Reads containing N-bases are also removed. (2) The pair-end reads are merged into a sequence according to their overlap relationship, and the minimum overlap length is 10 bp. (3) The maximum mismatch ratio allowed in the overlap area of the merged sequence is 0.2. (4) Distinguish samples and correcting the direction of the sequence based on the barcode and primer sequences at both ends of the sequence, the number of mismatches allowed in the barcode is 0, and the maximum number of mismatched primers is 2. And the sequence data was subsampled to equal sequencing depth for each sample. Operational taxonomic units (OTUs) with 97% similarity cutoff (Li et al., 2016) were clustered and chimeric sequences were identified and removed using UPARSE (version 7.1^1^). The taxonomy of each OTU representative sequence was analyzed by RDP Classifier^2^ against the 16S rRNA database (Silva 132/16S_bacteria) and ITS database (unite7.2/its_fungi) using confidence threshold of 0.7, relative abundance below 1% were combined as others.

### 3,3′-Diaminobenzidine and Nitro Blue Tetrazolium Staining

The content of H_2_O_2_ was estimated qualitatively in cucumber leaves using diaminobenzidine (DAB) staining and O^2–^ in the leaves was detected by nitro blue tetrazolium (NBT) staining (Kaur et al., 2016). Three leaves were detached and assayed for each treatment. To detect H_2_O_2_, leaves were dipped into DAB solution (1 mg/ml) prepared in double distilled water (pH = 3.8) in a Petri dish using tweezers. To detect O^2–^, the leaves were immersed in 6 mM NBT solution prepared in sodium citrate (pH = 6.0). Then dipped samples were vacuum infiltrated for 10 min at 60 KPa pressure and incubate at room temperature for 10 min under room light. After incubation, samples were boiled in absolute ethanol till chlorophyll is removed from samples completely. Finally, samples were cooled and stored in 20% glycerol. Images were captured using a light microscope.

### Assay of Antioxidases and MDA Concentration

Malondialdehyde (MDA) concentration of cucumber leaves was measured following methods described previously with some modifications (Hartman et al., 2004). Half a gram fresh leaves were homogenized in 5% (w/v) trichloroacetic acid (TCA) and the homogenate was centrifuged at 10,000 × *g* for 10 min. The supernatant was mixed with 5 ml of 0.5% thiobarbituric acid and heated at 100°C for 25 min, then was cooled to room temperature and centrifuged at 8,500 × *g* for 5 min. The absorbance was measured at 450, 532, and 600 nm. The MDA concentration was calculated using the formula: MDA = [6.45 (A_532_−A_600_) −0.56 A_450_]/fresh weight.

Superoxide dismutase (SOD) enzyme activity was determined following the method described previously (García-Limones et al., 2002). Briefly, 1 g fresh leaves were homogenized and centrifuged to prepare the supernatant. The crude enzyme extract was added into a reaction mixture to start the reaction and maintained under 4000 l× of light for 20 min. In the control reaction, the crude enzyme extract was replaced with phosphate buffer. The reaction was quenched by switching off the light, the absorbance at 560 nm was measured. The amount of enzyme that inhibited NBT reduction by 50% was defined as a unit of SOD activity. Results are expressed as units mg^–1^ of protein.

Peroxidase (POD) activity was assayed in a reaction mixture contained 2.9 ml of 0.05 M phosphate buffer, 0.5 ml of 2% H_2_O_2_, 0.1 ml of 2% guaiacol, and 0.1 ml of crude enzyme extract. The change of absorbance was measured at 470 nm. POD activity was defined as the amount of guaiacol oxidized per minute, and is expressed as nanomoles per minute per mg of protein.

Polyphenol oxidase (PPO) activity was measured following Tao et al. (2007) with some modifications. One gram fresh leaves were homogenized in phosphate buffer (pH 6.5) containing 0.20 g polyethylene glycol and 10 mM citric acid. The mixture was centrifuged at 10,000 × *g* for 15 min at 4°C. The activity assay was performed in a mixture containing 0.01 M substrate catechol, 0.1 M phosphate buffer and crude enzyme. Changes in absorbance at 420 nm were measured for 5 min. One unit of enzyme activity was defined as the change in absorbance at 420 nm for 1 g fresh weight per minute.

### GenBank Accession Number

The 16S ribosomal RNA sequence of *S. lydicus* M01 was deposited in NCBI under the GenBank accession numbers MN744679. The raw reads of Illumina MiSeq sequencing were deposited at NCBI’s Sequence Read Archive (SRA) database (accession number PRJNA598072).

### Statistical Analysis

Statistical analysis of the data was conducted by one-way ANOVA followed by Duncan’s test (*p* < 0.05) using SPSS 25.0 software.

## Results

### Isolation and Identification of the Pathogen Resistant Train *Streptomyces lydicus* M01

**FIGURE 1:**
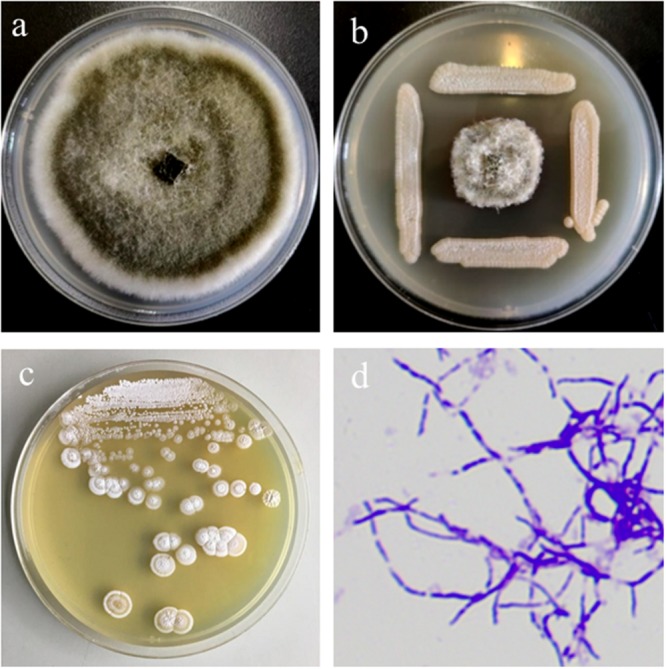
Antifungal activity of *Streptomyces lydicus* M01 against plant pathogen *Alternaria alternata* and morphological features of M01 colonies and mycelia. **(a)** Plant pathogen *A. alternata* grown on PDA plate as control. **(b)** Antagonistic effects of *S. lydicus* M01 against *A. alternata*. **(c)** Morphological features of the *S. lydicus* M01colonies. **(d)** Mycelia characteristics of *S. lydicus* M01 stained with crystal violet by light microscopy at 100× magnification.

With the supplement of cycloheximide and nalidixic acid, six bacterial isolates were isolated from the rhizosphere soil of Callery pear (*P. calleryana*). Among the isolated strains, M01 showed highest antifungal activity against the leaf blight plant pathogen *A. alternata* (Figures 1a,b). 16S rRNA gene analysis showed that M01 shared 99.93, 99.86, and 99.57% sequence identities with *S. lydicus* strain GS93 isolate 23, *S. lydicus* strain ATCC 25470 and *S. lydicus* strain NBRC 13058, respectively. Combined with morphological features of the colonies and mycelia (Figures 1c,d), M01 was classified as *S. lydicus* and the 16S rRNA gene sequence of M01 was submitted to GeneBank, the accession number is MN744679. And *S. lydicus* M01 was deposited in China general microbiological culture collection center (CGMCC NO: 16840). Neighbor-joining tree based on 16S ribosomal RNA showed that *S. lydicus* M01 formed a subclade with the nearest neighbor *S. lydicus* strain GS93, *S. lydicus* WYEC 108 and *S. lydicus* ATCC 25470 were separated in different clades (Supplementary Figure S1).

### Effect of *S. lydicus* M01 on the Growth of Cucumber Seedlings

**FIGURE 2:**
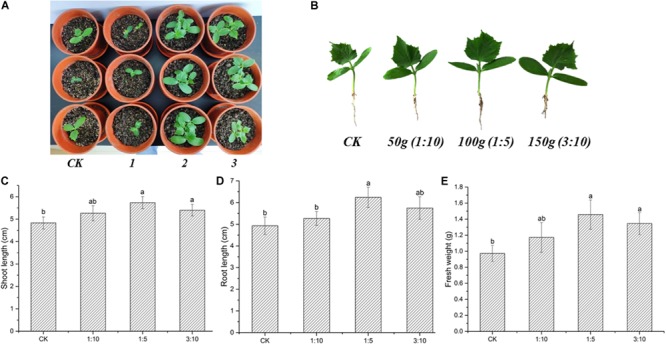
Effects of *S. lydicus* M01 on the growth of cucumber seedlings. **(A)** Effects of *S. lydicus* M01 on the cucumber seedlings growth in pot experiments. CK, control; 1, 2, and 3, *S. lydicus* M01 culture were mixed with the soil at a ratio of 1:10, 1:5, and 3:10 (w:w), respectively. **(B)** Effects of *S. lydicus* M01 on phenotype of cucumber seedlings under different inoculum amount. **(C)** Shoot length, **(D)** root length and **(E)** fresh weight of cucumber plants grown in soil treated or not (CK) with *S. lydicus* M01. Statistical analysis was conducted by one-way ANOVA followed by Duncan’s test (*p* < 0.05) using SPSS 25.0 software. Different letters on columns indicate significant difference (*p* < 0.05).

The effects of *S. lydicus* M01 inoculation on plant growth promotion were investigated in cucumber plants. After 21 days of inoculation, growth promotion effects on cucumber plants treated with 50 ml M01 culture were not significant. However, when the inoculation amount was increased to 100 ml, the plant height, root status and leaf number and expansion of cucumber seedlings were all significantly enhanced compared to the CK (Figures 2A,B). Further increase in the inoculation amount reduced the growth promotion effects. With the treatment of 100 ml M01 culture, the shoot length, root length, and fresh weight were increased by 18.71, 26.53, and 49.74%, respectively, compared to CK (Figures 2C–E).

To characterize of plant growth promoting traits of *S. lydicus* M01, the IAA production, phosphate solubilization potential, siderophore production and ACC deaminase activity were determined. The IAA produced was determined to be 19.81 ± 1.7 μg/ml. The presence of a clear zone around the colonies indicated that *S. lydicus* M01 has the ability to solubilize phosphate and produces siderophore (Supplementary Figure S2). ACC deaminase activity was also qualitatively evaluated, successful growing on the MM agar supplemented with ACC indicated ACC deaminase activity.

### Effects of *S. lydicus* M01 on Soil Microbial Community Composition

In this study, analysis based on Illumina sequencing of the six rhizosphere soil samples generated 289,648 16S rRNA gene sequences and 407,987 fungal ITS sequences. The average amplicon length of the six bacterial samples was 414 bp, the average amplicon length of the six eukaryotic samples was 229 bp. After quality trimming, an average of 39,747 reads per sample was generated for the bacteria analyses and 67,544 reads per sample for the eukaryotic sample. The subsampling depth was equalized to the depth of the smallest sample (32,213 reads for the bacteria analyses and 56,672 reads for the eukaryotic sample). A total of 1864 OTUs for bacteria and 623 OTUs for fungi were identified. OTUs with abundance greater than 1% were defined as dominant, while OTUs with abundance less than 1% were combined as “others.”

**FIGURE 3:**
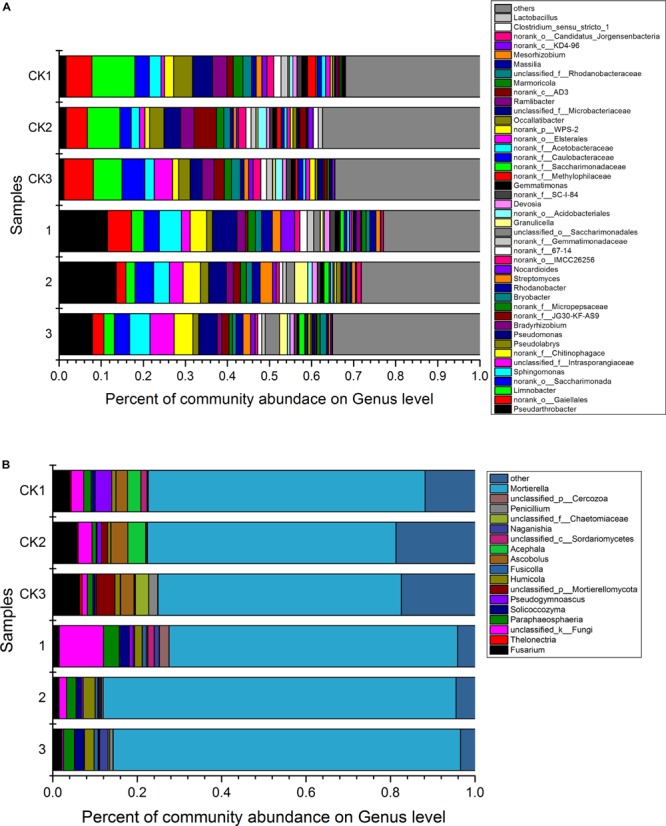
The relative abundance of dominant bacterial genera **(A)** and fungal genera **(B)** for soil samples collected from soil treated or not with *Streptomyces lydicus* M01. CK1, CK2, and CK3, untreated plants; 1,2, and 3, treated plants. The relative abundance was based on the proportional frequencies of DNA sequences that could be classified at the genus level. The different numbers (1, 2, and 3) indicate the three replications.

**FIGURE 4:**
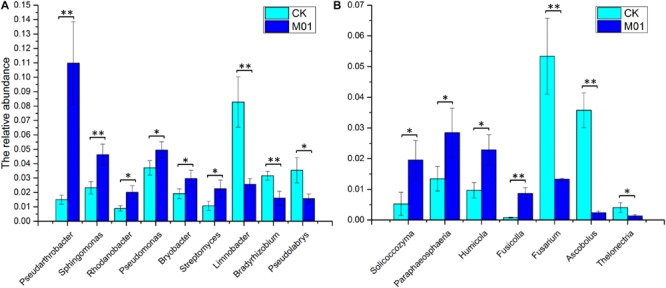
The relative abundance of the main bacterial **(A)** and fungal genera **(B)** with significant differences for the soil samples collected from soil treated or not with *S. lydicus* M01. CK, untreated plants; M01, treated plants. Each histogram represents the mean ± SE of three independent experiments. An independent sample *t*-test was implemented for statistical analysis of the significance between the M01-treated and the CK group (* for 0.01 < *p* < 0.05; ** for *p* < 0.01).

Analysis against 16S rRNA database and ITS database revealed that 43 dominant bacterial and 17 dominant fungal genera were identified at the genus level in samples from different treatments (Figure 3). The 43 dominant bacterial genera included *Pseudarthrobacter*, *Limnobacter*, *Sphingomonas*, *Pseudolabrys*, *Pseudomonas*, *Bradyrhizobium*, *Bryobacter*, *Rhodanobacter*, *Streptomyces*, *Nocardioides*, *Granulicella*, *Devosia*, *Gemmatimonas*, *Occallatibacter*, *Ramlibacter*, *Marmoricola*, *Massilia*, *Mesorhizobium*, *Lactobacillus* and 24 unclassified bacteria (Figure 3A). Analysis based on the relative abundance of classified bacterial genera revealed that treatment of *S. lydicus* M01 influenced the bacterial and fungal composition of the soil compared with non-treated soil. The abundance of species increased included *Pseudarthrobacter, Sphingomonas*, *Rhodanobacter*, *Pseudomonas*, *Bryobacter*, and *Streptomyces*, the abundance of species decreased included *Limnobacter*, *Bradyrhizobium*, and *Pseudolabrys* (Figure 4A and Supplementary Table S1). The 17 dominant fungal genera included *Mortierella*, *Penicillium*, *Naganishia*, *Acephala*, *Ascobolus*, *Fusicolla, Humicola*, *Pseudogymnoascus*, *Solicoccozyma*, *Paraphaeosphaeria*, *Thelonectria*, *Fusarium* and five unclassified fungi (Figure 3B). The species *Mortierella* occupied a large portion in the total population in all samples, and the abundance was significantly increased in the treated samples. Except for *Mortierella*, the abundance of several other species such as *Solicoccozyma*, *Paraphaeosphaeria*, *Humicola*, and *Fusicolla* were also enriched. In the contrast, the abundance of *Fusarium*, *Ascobolus*, and *Thelonectria* which contain potential fungal pathogen species were significantly reduced (Figure 4B and Supplementary Table S1).

### *Streptomyces lydicus* M01 Alleviates Foliar Disease Caused by *Alternaria alternata*

In contrast to soil microorganisms, microbial resources from the plant phyllosphere that have the potential to suppress diseases and support plant growth are limited (Rastogi et al., 2013). Therefore, it is tempting to take advantage of soil-borne microbes to develop foliar treatments of crops to fight economically relevant diseases. Thus, *S. lydicus* M01’s effects on foliar disease caused by *A. alternata* were investigated.

**FIGURE 5:**
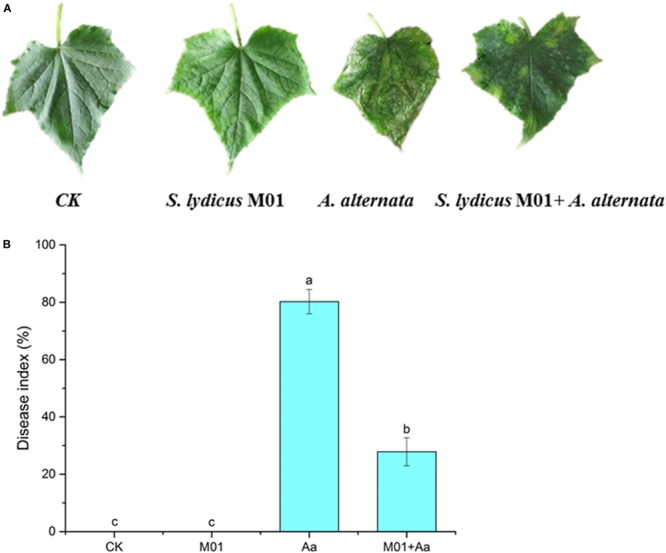
Symptom **(A)** and disease index **(B)** on cucumber leaves 30 days after inoculation with *A. alternata* following different treatments. CK, untreated plants; *S. lydicus* M01, plants treated with *S. lydicus* M01; *A. alternata*, plants treated with *A. alternata*; *S. lydicus* M01+ *A. alternata*, plants pretreated with *S. lydicus* M01 then with *A. alternata*. The histogram represents the mean ± SE of three independent experiments. Statistical analysis was conducted by one-way ANOVA followed by Duncan’s test (*p* < 0.05) using SPSS 25.0 software. Different letters on columns indicate significant difference (*p* < 0.05).

**FIGURE 6:**
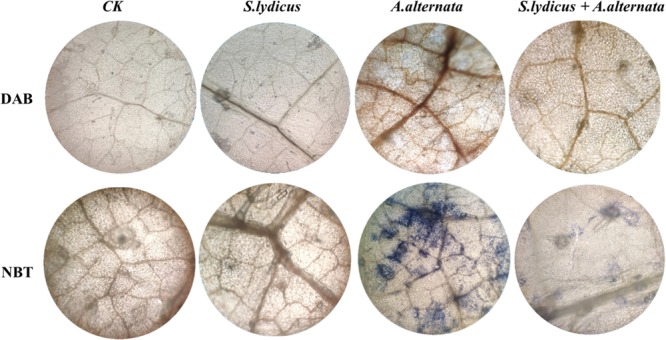
Analysis of ROS accumulation in cucumber leaves following different treatments. DAB, H_2_O_2_ accumulation was detected by DAB staining. NBT, O^2–^ accumulation was detected by NBT staining.

After inoculating with *A. alternata*, disease symptoms appeared on cucumber plants including yellow and wilted leaves. The disease incidence reached 80.25%. However, pretreatment with *S. lydicus* M01 significantly reduced disease incidence, with less yellowing and wilting of the cucumber leaves (Figure 5A). Compared to the control, the application *S. lydicus* M01 reduced the disease incidence by 52.4% (Figure 5B and Supplementary Table S2). ROS were postulated to be an integral part of the defense response of the plant, which are involved in the interaction of plants with pathogenic fungi. In this study, the production of hydrogen peroxide in leaves was detected by DAB staining, and the superoxide accumulation was analyzed by NBT staining. Leaves of plants treated with *A. alternata* showed the deepest brown color than control and other treatments, however, leaves of plants pretreated with *S. lydicus* M01 showed lighter brown color after the perception of pathogens. Similarly, blue staining was observed in leaves of plants treated with *A. alternata*, but pretreatment of *S. lydicus* M01 significantly reduced the staining intensity (Figure 6 and Supplementary Figure S4).

**FIGURE 7:**
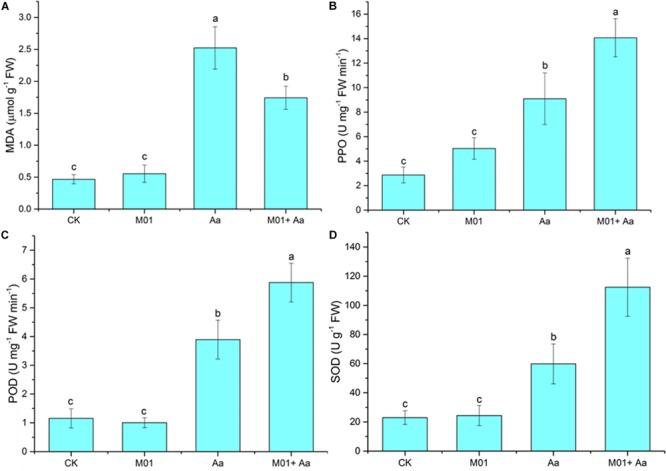
Activities of defense-related enzyme and concentrations of malondialdehyde measured in leaves. **(A)** Malondialdehyde (MDA). **(B)** Polyphenol oxidase (PPO). **(C)** Peroxidase (POD). **(D)** Superoxide dismutase (SOD). Statistical analysis was conducted by one-way ANOVA followed by Duncan’s test (*p* < 0.05) using SPSS 25.0 software. Different letters on columns indicate significant difference (*p* < 0.05).

Malondialdehyde content is considered as an index of membranous lipid peroxidation (Morsy et al., 2007) and stress-related enzymes PPO, POD, and SOD are commonly used to assess the physiological and biochemical responses of plants to biotic stresses (Gechev et al., 2003). The MDA content of plant leaves treated with *A. alternata* was significantly increased compared to the control, but leaves of plant pretreated with *S. lydicus* M01 was lower (Figure 7A). A significant increase in the PPO activity was also found in plant treated with the plant pathogen, but a higher activity was produced with the pretreatment of *S. lydicus* M01 (Figure 7B). Similar trends were observed in the POD and SOD activity determination, the highest activity was found in the plant treated with *S. lydicus* M01 then the plant pathogen (Figures 7C,D). Notably, an increase of PPO activity was observed in plant treated with *S. lydicus* M01 without invasion of plant pathogen.

## Discussion

The green revolution of the past century has significantly improved crop yield by extensive use of mineral fertilizers and chemically synthesized pesticides. Despite immediate benefits, this strategy is reaching its limits in maintaining food security for a growing population (Bosch et al., 2014). Alternative strategies for plant growth promotion and protection against pests are associated with soil microbes for nutrition gains and disease suppression (Rey and Schornack, 2013). Among the various soil-borne bacteria and fungi associated with agriculture, species in the *Streptomyces* genus, which has been highlighted in medical drug discoveries, are major actors in soil suppression of diseases (Cordovez et al., 2015; Cha et al., 2016) owing to the numerous metabolites they produce which inhibit the development of microbial pathogens. Despite their popularity as biocontrol agents, understanding of *Streptomyces*’s function as biological fertilizers is limited, especially their influence in soil microbial community.

In the present study, *S. lydicus* M01 distributed in the rhizosphere soil of Callery pear (*P. calleryana*) was isolated and exhibited strong activity against leaf blight plant pathogen *A. alternata*. Phylogenetic analysis revealed that *S. lydicus* M01 was clustered in a different clade compared with the commercialized *S. lydicus* WYEC 108 (Yuan and Crawford, 1995) and other *Streptomyces* strains, which indicated potential differences. In addition, comparative genomic analyses showed that closely related *Streptomyces* strains have high diversity in the secondary metabolite gene clusters they contain (Hwang et al., 2014; Jia et al., 2017). In the pot experiments, the application of *S. lydicus* M01 had a positive effect on the growth of cucumber seedlings, compared to the CK treatment, the plant height, fresh weight, and root status were all increased to different degrees. This result is in agreement with our previous study when *S. lydicus* M01 promoted cucumber seedlings growth in hydroponic experiments (Supplementary Figure S3). These results, in combination with the production of IAA, ACC desaminase, and siderophores demonstrate that *S. lydicus* M01 has the capacity to stimulate plant growth. Then, the underlying mechanism was investigated in the aspect of changes in soil microbial community following the application of *S. lydicus* M01. To minimize error caused by microbe culture, all cell suspension used in the treatment were from the same batch and sterile nutrient broth of the same composition were used as control. Thus, their influence the microbial community should be minor.

Analysis of microbial community composition and structure revealed that a higher relative abundance of *Pseudarthrobacter*, *Sphingomonas*, *Rhodanobacter*, *Pseudomonas*, *Bryobacter*, and *Streptomyces* genera were identified in the *S. lydicus* M01 treated soil. Species from *Pseudarthrobacter* are capable of degrading high concentrations of environmental contaminants such as 4-chlorophenol and phenanthrene (Westerberg et al., 2000; Kim et al., 2008). As such, they were used in bioremediation. *Sphingomonas* are widely distributed in nature, having been isolated from plant root systems and other sources. Reports showed that *Sphingomonas* stimulates the growth plants through phytohormone production, nitrogen fixation and driving developmental plasticity in the roots (Yang et al., 2014; Luo et al., 2019). Additionally, *Sphingomonas* was also shown to alleviate salinity stress in plants (Khan et al., 2017). *Rhodanobacter* are known denitrifying bacteria that are used for bioremediation (Prakash et al., 2012), interestingly, several strains showed biological control activity toward root-rot plant pathogen (De Clercq et al., 2006). *Pseudomonas* is one of the most common and widely used biocontrol and PGPR strains due to their pathogen inhibition and systemic resistance inducing properties (Chin-A-Woeng et al., 2000; Haas and Défago, 2005). *Bryobacter* inhabit in peat, but the function is unknown (Kulichevskaya et al., 2010). The relative abundance of *Streptomyces* was also significantly increased 21 days after inoculation which may indicate an effective colonization of *S. lydicus* M01. Most of the bacteria from the genera listed above have characteristics associated with pathogen inhibition, plant auxin biosynthesis or bioremediation. However, the abundance of *Bradyrhizobium* and *Pseudolabrys* that both function in nitrogen fixation (Hennecke, 1990; Rangel et al., 2017; Wongdee et al., 2018) were significantly reduced. Furthermore, the abundance of *Limnobacter* species that have the ability to oxidize thiosulfate (Lu et al., 2011; Vedler et al., 2013) was also reduced. Overall, most of the bacterial communities significantly altered by *S. lydicus* M01 treatment had a positive effect on growth promotion, biological control, alleviation of abiotic stress, or bioremediation.

The use of *S. lydicus* M01 in the plant growth promotion of cucumber seedlings occurs not only through shifting the rhizospheric bacterial community but also through altering the composition of the fungal community. Previous studies showed that *Fusicolla* sp. is a putative plant-growth-promoting rhizobacteria that correlated positively with canola yield (Lay et al., 2018) and *Humicola* is a fungus with potential for biological control of plant diseases (Ko et al., 2011). In this study, abundance of the two species were significantly increased in the presence of *S. lydicus* M01. Furthermore, the fungal genera *Solicoccozyma* and *Paraphaeosphaeria* were also enriched in comparison with the control soil. *Solicoccozyma* are yeasts isolated from soil that function in biodegradation (Stosiek et al., 2019). Species in *Paraphaeosphaeria* were found to enhance plant temperature tolerance in Arabidopsis (McLellan et al., 2007), thus the enrichment of *Paraphaeosphaeria* may contribute to the alleviation of abiotic stress of cucumbers. Notably, *Fusarium* was identified in the all soil samples and its abundance was significantly reduced in the presence of *S. lydicus* M01. Since this fungus is widely distributed in soil and most species are harmless, plant pathogenic species were not considered in the present study. Some species of *Ascobolus* were found on rotting *Brassica* stems (Buczacki and Wilkinson, 1992) and *Thelonectria* was reported to produce glycosidase inhibitors (Miyauchi et al., 2016), both genera were decreased in the presence of *S. lydicus* M01. Plants are tightly associated with complex microbial communities, modification of the plant microbiome play an important role to reduce the occurrence of plant disease, increase agricultural production, and reduce chemical inputs. Thus, overall shift in soil microbial community by M01 or other microbes is important in agriculture, resulting in more sustainable agricultural practices.

Plant growth-promoting rhizobacteria affect plant growth in direct and indirect ways. The indirect promotion of plant growth occurs when PGPR decrease the deleterious effects of phytopathogenic organisms or ISR. Foliar disease caused by *A. alternata* develops on leaves and often spread through air. However, colonization of PGPR on the aerial parts of plants remains a technical bottleneck (Samac et al., 2003). Thus, in this study, the alleviation of foliar disease by *S. lydicus* M01 around the cucumber rhizosphere reflected indirect effects of PGPR on plant growth promotion and disease suppression. The ROS production is one of the earliest cellular responses following successful pathogen recognition and accumulation of ROS were consistently observed in the plant after the perception of pathogens (Torres et al., 2006). However, excessive production of ROS causes progressive oxidative damage and ultimately cell death (Das and Roychoudhury, 2014). The reduced staining intensity of ROS in cucumber leaves indicated that the *S. lydicus* M01 treatment reduced ROS accumulation. Further investigation revealed that antioxidants related with ROS scavenging were influenced by *S. lydicus* M01 treatment under biotic stress. MDA content is usually increased under stressed conditions such as pathogenic infection (Heber et al., 1996). In this study, pretreatment with *S. lydicus* M01 significantly reduced the MDA content, which reflects a lower level of membranous lipid peroxidation. This result agrees with previous report that the content of MDA in leaves treated with biological fertilizer was much lower than the control plants (Wu et al., 2008). Antioxidases such as SOD and POD are associated with eliminating ROS (Asada, 1992), whereas PPO may be induced to produce lignin and other phenolics to strengthen the cell walls (Avdiushko et al., 1993). In this study, the POD, SOD, and PPO enzyme activities were increased significantly when plants were pretreated with *S. lydicus* M01 before the invasion of plant pathogen, but the activities of these enzymes increased less in cucumber only treated with *A. alternata*. Similar results were also observed in previous report when antagonistic bacterium *B. subtilis* strain AR12 was used to induce defense enzymes toward *Ralstonia solanacearum* in tomato (Li et al., 2008). These results suggest that *S. lydicus* M01 enhanced disease resistance of cucumber, at least in part, by increasing activities of defense related enzymes. Further studies are required for investigation of ISR and related signaling pathways.

## Conclusion

In conclusion, the application of *S. lydicus* M01 effectively promoted the plant growth and suppressed foliar disease caused by *A. alternata* in cucumbers. *S. lydicus* M01 may systemically affect plant-soil interactions by producing auxins, siderophores and ACC deaminase and increasing the abundance of certain plant beneficial microbes, such as bacteria genera *Pseudarthrobacter*, *Sphingomonas*, *Rhodanobacter*, and *Pseudomonas* and fungi genera *Fusicolla* and *Humicola*. Furthermore, *S. lydicus* M01 enhanced plant disease resistance through induced antioxidases activity, possibly together with other beneficial microbes influenced around rhizosphere. The beneficial plant microbiome interactions represent a promising sustainable solution to improve agricultural production instead of chemical fertilizers.

## Data Availability Statement

The 16S ribosomal RNA sequence of *Streptomyces lydicus* M01 was deposited in NCBI under the GenBank accession numbers MN744679. The raw reads of Illumina MiSeq sequencing were deposited at NCBI’s Sequence Read Archive (SRA) database (accession number PRJNA598072).

## Author Contributions

MW, JX, and JM performed the experiments. MW analyzed the sequencing data. XF provided the technical assistance. HX and MW designed the experiments. MW wrote the manuscript. HX and HY revised the manuscript.

## Conflict of Interest

The authors declare that the research was conducted in the absence of any commercial or financial relationships that could be construed as a potential conflict of interest.

## Footnotes

1 http://drive5.com/uparse/

2 http://rdp.cme.msu.edu/
