# Supplementary material for: Streptomyces lydicus M01 Regulates Soil Microbial Community and Alleviates Foliar Disease Caused by Alternaria alternata on Cucumbers
Source: Front Microbiol. 2020 May 15;11:942. doi: 10.3389/fmicb.2020.00942 (PMC7243425; doi:10.3389/fmicb.2020.00942)
Supplement: Supplementary file 1 [file Data_Sheet_1.docx]

Supplementary Material

## Supplementary Figures


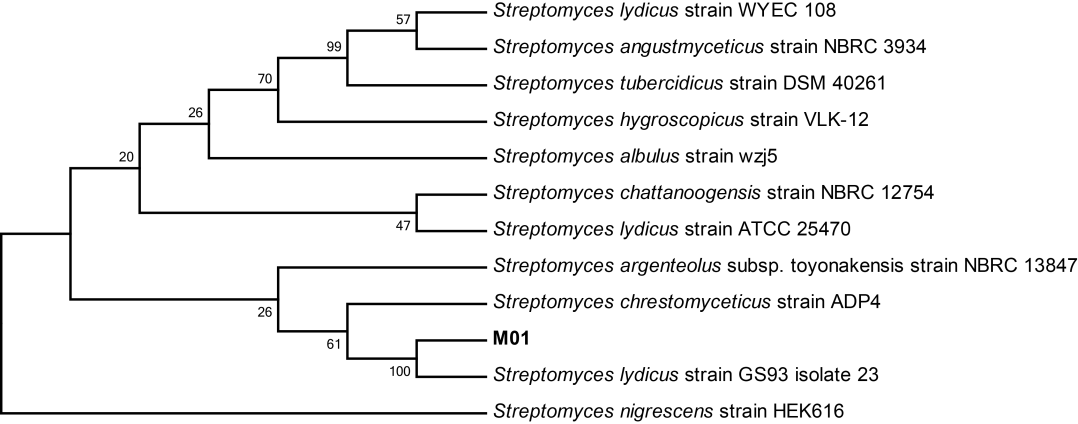


**Figure S1** Neighbor-joining tree based on 16S ribosomal RNA showing the relationship among M01 and other *Streptomyces* species. Gaps were treated using the method of pairwise deletion and the evolutionary distances were computed using the Maximum Composite Likelihood method.

**
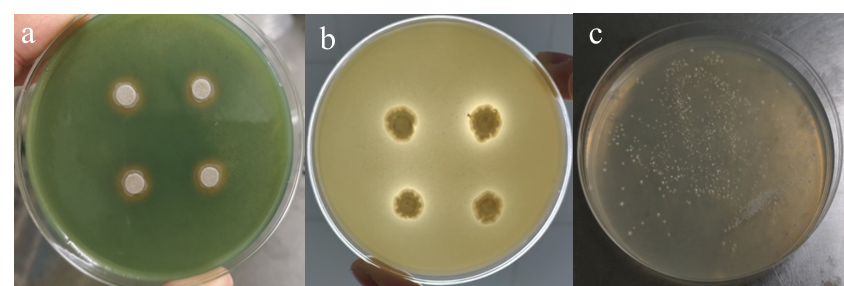
**

**Figure S2** Growth promoting characteristics of *S. lydicus* M01. (a) Siderophore production; (b) phosphate solubilization; (c) ACC deaminase activity.


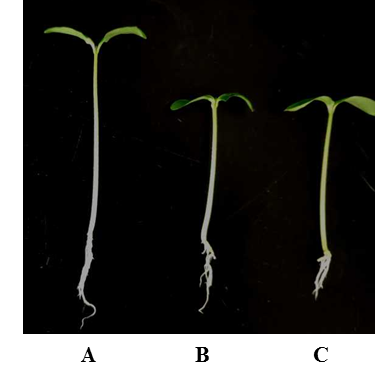


**Figure S3** Effects of *S. lydicus* M01 on the cucumber seedlings growth in hydroponic experiments (A) Treatment with *S. lydicus* M01; (B) Treatment with sterile distilled water as control; (C) Treatment with sterile broth as control.


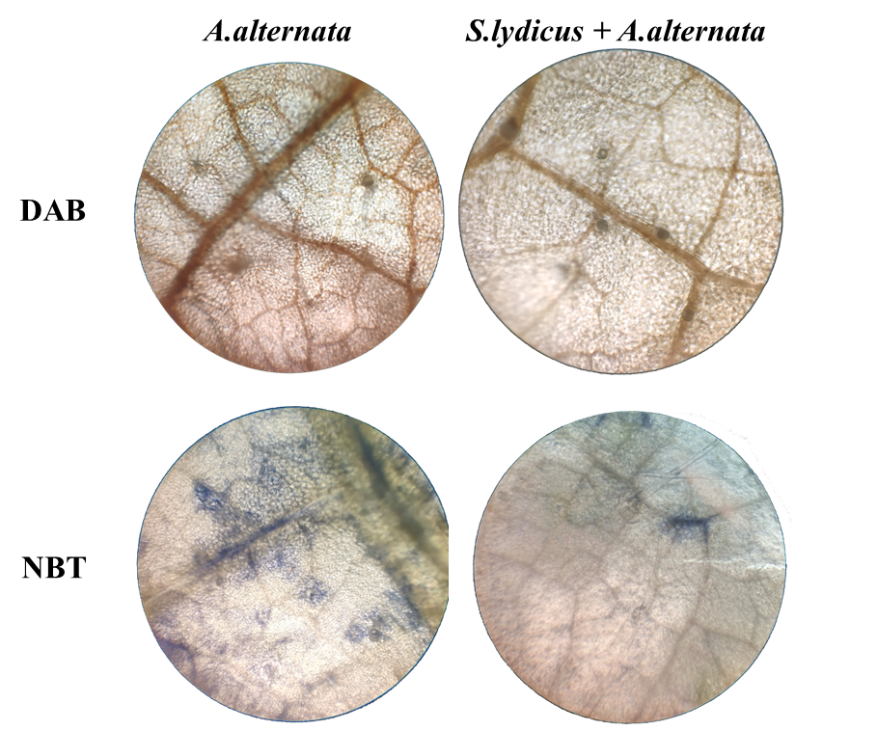


**Figure S4** Additional replicate photos for analysis of ROS accumulation in cucumber leaves following diﬀerent treatments. DAB：H_2_O_2_ accumulation was detected by DAB staining. NBT: O^2-^ accumulation was detected by NBT staining.

**Table S1** The actual data point of relative abundance for each replicate in Figure 4.

| Genera | CK1 | CK2 | CK3 | M01_1 | M01_2 | M01_3 |
| --- | --- | --- | --- | --- | --- | --- |
| *Pseudarthrobacter* | 0.01667 | 0.01704 | 0.01125 | 0.11481 | 0.13567 | 0.07875 |
| *Sphingomonas* | 0.02800 | 0.01953 | 0.02241 | 0.05234 | 0.03828 | 0.04827 |
| *Rhodanobacter* | 0.01093 | 0.00720 | 0.00860 | 0.02527 | 0.01841 | 0.01670 |
| *Pseudomonas* | 0.04165 | 0.03805 | 0.03167 | 0.05587 | 0.04476 | 0.04772 |
| *Bryobacter* | 0.02046 | 0.01530 | 0.02166 | 0.03562 | 0.02439 | 0.02913 |
| *Streptomyces* | 0.01376 | 0.00730 | 0.01107 | 0.02014 | 0.02950 | 0.01807 |
| *Limnobacter* | 0.10232 | 0.07742 | 0.06868 | 0.02987 | 0.02182 | 0.02524 |
| *Bradyrhizobium* | 0.03427 | 0.03229 | 0.02831 | 0.02043 | 0.01698 | 0.01087 |
| *Pseudolabrys* | 0.04473 | 0.03409 | 0.02741 | 0.01368 | 0.01943 | 0.01422 |
| *Solicoccozyma* | 0.0094 | 0.0020 | 0.0044 | 0.0233 | 0.0123 | 0.0231 |
| *Paraphaeosphaeria* | 0.0176 | 0.0097 | 0.0130 | 0.0374 | 0.0221 | 0.0260 |
| *Humicola* | 0.0097 | 0.0072 | 0.0122 | 0.0182 | 0.0280 | 0.0224 |
| *Fusicolla* | 0.0007 | 0.0006 | 0.0010 | 0.0099 | 0.0065 | 0.0096 |
| *Fusarium* | 0.0397 | 0.0569 | 0.0636 | 0.0135 | 0.0131 | 0.0206 |
| *Ascobolus* | 0.0268 | 0.0398 | 0.0318 | 0.0029 | 0.0018 | 0.0023 |
| *Thelonectria* | 0.0033 | 0.0030 | 0.0059 | 0.0015 | 0.0017 | 0.0009 |

CK: untreated plants. M01: treated plants.

**Table S2** The actual data point of disease index for each replicate in Figure 5B.

| Treatment | 1 | 2 | 3 |
| --- | --- | --- | --- |
| CK | 0 | 0 | 0 |
| M01 | 0 | 0 | 0 |
| Aa | 84.56 | 76.04 | 80.15 |
| M01+Aa | 26.76 | 33.2 | 23.61 |
